# Specialist trainees on rotation cannot replace dedicated consultant clinicians for antimicrobial stewardship of specialty disciplines

**DOI:** 10.1186/2047-2994-1-36

**Published:** 2012-11-17

**Authors:** Chay Leng Yeo, Jia En Wu, Gladys Wei-Teng Chung, Douglas Su-Gin Chan, Dale Fisher, Li Yang Hsu

**Affiliations:** 1 Department of Pharmacy, National University Health System, 5 Lower Kent Ridge Road, Singapore, 119074, Singapore; 2 Department of Laboratory Medicine, National University Health System, 5 Lower Kent Ridge Road, Singapore, 119074, Singapore; 3 Department of Medicine, National University Health System, NUHS Tower Block Level 10, 1E Kent Ridge Road, Singapore, 119228, Singapore; 4 Saw Swee Hock School of Public Health, National University of Singapore, MD3, 16 Medical Drive, Singapore, 117597, Singapore

**Keywords:** Antimicrobial stewardship, Antimicrobial resistance, Trainee supervision, Compliance

## Abstract

Our prospective-audit-and-feedback antimicrobial stewardship (AS) program for hematology and oncology inpatients was switched from one led by dedicated clinicians to a rotating team of infectious diseases trainees in order to provide learning opportunities and attempt a “de-escalation” of specialist input towards a more protocol-driven implementation. However, process indicators including the number of recommendations and recommendation acceptance rates fell significantly during the year, with accompanying increases in broad-spectrum antibiotic prescription. The trends were reversed only upon reverting to the original setup. Dedicated clinicians play a crucial role in AS programs involving immunocompromised patients. Structured training and adequate succession/contingency planning is critical for sustainability.

## Letters to the Editor

Sir,

Effective antimicrobial stewardship programs (ASPs) have been shown to reduce inappropriate antimicrobial prescription and healthcare costs [1]. However, antimicrobial stewardship per se is loosely defined, and the field includes multiple strategies and interventions that are implemented in healthcare settings of diverse complexity [1,2]. There are few formal training curricula for antimicrobial stewardship practitioners currently, although their importance has been highlighted repeatedly [1]. Most antimicrobial stewardship practitioners have instead undergone experiential learning during the establishment of their ASPs.

We had previously shown that a safe and effective prospective audit and feedback ASP led by dedicated consultant-level clinicians (an infectious diseases physician and a clinical microbiologist) could be implemented even in the complex setting of hematology and oncology inpatients within a tertiary university hospital, with broad-spectrum antibiotics reviewed on the 4^th^ and 7^th^ day of prescription [3]. In order to improve manpower utilization, provide learning opportunities for trainees, and to determine if “de-escalation” of specialist input towards a more protocol-driven implementation was possible, the ASP was switched to a team of four infectious diseases (ID) trainees rotating weekly from August 2010, before being reverted to the original consultant clinicians from August 2011 because of suboptimal results. The pharmacist remained the same throughout all three periods. We present the impact of this personnel change on the ASP’s intervention outcomes and the antimicrobial utilization among hematology-oncology inpatients across 3 years (August 2009 to July 2012) below.

All data were captured prospectively as part of an ongoing audit of the ASP, and part of the first year’s data had been published earlier [3]. The antibiotic prescriptions reviewed include all carbapenems, 3^rd^- and 4^th^-generation cephalosporins, intravenous β-lactam/ β-lactamase inhibitors, and vancomycin among others; and the ASP’s system has previously been described, with compliance to recommendations recorded within 48 hours [3]. Patients with accepted recommendations were assessed for clinical outcomes at one week post-acceptance [3].

The results of the audit are shown in Table 1, where Year 2 was the period where ID trainees were rotated through the ASP. Carbapenems (28.2%), piperacillin/tazobactam (26.7%) and cephalosporins (19.4%) formed the majority of the prescriptions reviewed by the ASP pharmacist, and a total of 1,569 recommendations were made during this period. Recommendation and recommendation acceptance rates were significantly higher in Years 1 and 3 compared to Year 2. The prescription of all intravenous antibiotics, carbapenems and vancomycin increased significantly in Year 2, but decreased in Year 3; piperacillin/tazobactam prescription significantly increased across all 3 years, whereas 3^rd^- and 4^th^-generation cephalosporin prescription fell in Year 2.

**Table 1:** Results of the ASP audit on recommendations and outcomes over 3 years (August 2009 to July 2012)

	**Year 1**	**Year 2**	**Year 3**
	**(August 2009 to July 2010)**	**(August 2010 to July 2011)**	**(August 2011 to July 2012)**
Number of prescriptions reviewed	1,414	1,168	1,567
Number of antimicrobial stewardship recommendations made (percentage of prescriptions reviewed)	649 (45.9%)	363 (31.1%)^b^	557 (35.5%)
• Discontinuation of antibiotic therapy	239	135	227
• De-escalation of antibiotic therapy	198	74	176
• Intravenous to enteral switch	49	10	47
• Escalation of antibiotic therapy	25	7	13
• Antibiotic dosing	39	68	49
• Others^a^	99	69	45
Recommendation acceptance (percentage of recommendations accepted)	570 (87.7%)	266 (73.3%)^b^	464 (83.3%)
Antimicrobial prescription (defined daily dose/1,000 inpatient-days)			
• All intravenous antibiotics	468.6	580.3^c^	513.7
• All carbapenems	130.8	181.2^c^	149.1
• 3^rd^ and 4^th^-generation cephalosporins	128.2	95.1^d^	108.7
• Piperacillin/tazobactam	62.2	116.0^c^	160.8
• Vancomycin	80.9	108.7^e^	38.1

^a^”Others” include advice on duration of antibiotics, further investigations, and advice on referral to infectious diseases specialists.

^b^Statistically significant difference between Years 1 and 2, and Years 2 and 3, based on the chi-square test.

^c^Statistically significant difference between Years 1 and 2, and Years 2 and 3, based on Wilcoxon rank-sum test.

^d^Statistically significant difference between Years 1 and 2 only, based on the Wilcoxon rank-sum test.

^e^Statistically significant difference between Years 2 and 3 only, based on the Wilcoxon rank-sum test.

Improved patient clinical outcomes – defined as resolution of clinical signs and laboratory markers of infection 7 days post-acceptance of ASP recommendations – was observed in 79.2%, 68.4% and 66.0% of patients in Years 1, 2, and 3 respectively. The majority of patients with no clinical improvement either had no infection or had advanced malignancies for which active management was deemed futile. Only a small proportion (3.1%) of patients deteriorated post-acceptance of ASP recommendations as a consequence of worsening sepsis or new onset nosocomial infection.

Our experience highlights several pertinent issues for prospective audit and feedback ASPs. Firstly, dedicated clinician specialists appear more effective than unsupervised trainee ID physicians in complex medical disciplines despite the often-algorithmic nature of ASP work. A random retrospective audit of 100 ASP-reviewed prescriptions in Year 2 (Table 2) revealed a significant number of cases where recommendations were not issued despite local antibiotic guidelines being flouted [4] or where antibiotics could be de-escalated in view of microbial susceptibility results. De-escalation of consultant presence probably resulted in trainees behaving more conservatively during ASP sessions, particularly for patients with persistent febrile neutropenia. The lower recommendation acceptance rates likely occurred because the primary physicians had more difficulty accepting recommendations from trainees rather than consultants (all recommendations were signed and included a name stamp), and the weekly rotation of trainees resulted in conflicting recommendations to the primary teams on occasion, reducing credibility in the ASP.

**Table 2:** Results of a random retrospective audit of 100 ASP-reviewed prescriptions in Year 2

	**Number of events**
Antibiotics reviewed^a^	
• Carbapenems	37
• 3^rd^ and 4^th^ generation cephalosporins	17
• Piperacillin/tazobactam	32
• Vancomycin	16
• Others	14
Number of antimicrobial stewardship recommendations made:	28
• Discontinuation of antibiotic therapy (percentage of all recommendations)	12 (42.9%)
• De-escalation of antibiotic therapy (percentage of all recommendations)	5 (17.9%)
• Intravenous to enteral switch (percentage of all recommendations)	1 (3.6%)
• Escalation of antibiotic therapy (percentage of all recommendations)	1 (3.6%)
• Antibiotic dosing (percentage of all recommendations)	3 (10.7%)
• Others^b^ (percentage of all recommendations)	6 (21.4%)
Recommendation acceptance (percentage of all recommendations):	20 (71.4%)
• Discontinuation of antibiotic therapy (percentage of similar recommendations)	9 (75.0%)
• De-escalation of antibiotic therapy (percentage of similar recommendations)	3 (60.0%)
• Intravenous to enteral switch (percentage of similar recommendations)	0 (0%)
• Escalation of antibiotic therapy (percentage of similar recommendations)	1 (100%)
• Antibiotic dosing (percentage of similar recommendations)	3 (100%)
• Others^b^ (percentage of similar recommendations)	4 (66.7%)
Potential additional antimicrobial stewardship recommendations based on local guidelines [4] and/or clinical criteria:	
• Discontinuation of antimicrobial therapy^c^	3
• De-escalation of antibiotic therapy^d^	5
• Intravenous to enteral switch	3

^a^16 prescriptions involved 2 different antibiotics.

^b^”Others” include advice on further investigations (1 case), advice on referral to infectious diseases specialists (2 cases), and discontinuation of concurrent antibiotics (3 cases).

^c^Treatment of asymptomatic bacteriuria (1 case), continuation of antibiotics after resolution of occult febrile neutropenia (2 cases).

^d^Oral antibiotics for low-risk febrile neutropenia (2 cases), use of narrower-spectrum antibiotics for culture-positive infections (3 cases).

However, simultaneously providing rotational consultant oversight would not have resolved all issues, particularly those of conflicting opinions [5], and lack of ownership. In reality, only a fraction of ID specialists elect to participate in antimicrobial stewardship, and most trainees and consultants face major challenges in making therapeutic recommendations without direct patient contact.

The results for cephalosporins and piperacillin/tazobactam are consistent with local guidelines recommending the switch from ceftazidime to piperacillin/tazobactam for febrile neutropenia in late 2009 [4], with consequent “spillover” to antibiotic prescription for other nosocomial infections. This again highlights the difficulty in reducing the prescription of “workhorse” antibiotics in ASPs where reviews occur only on the 4^th^ day of prescription [3].

In conclusion, dedicated consultant-level clinicians play a crucial role in prospective audit and feedback ASPs, especially in a setting with immunocompromised patients. In situations where manpower limitations are unavoidable, employment of alternative strategies such as formulary restriction – where outcomes are less provider-dependent – may improve resource utilization [1]. However, it would be better to have structured antimicrobial stewardship training programs for trainee ID physicians and to have adequate succession/contingency planning.

## Competing interests

LYH has received research funding and speaker’s honoraria from Pfizer, AstraZeneca, Janssen & Cilag, and Merck, Sharpe & Dohme. Other authors have no conflicts of interest to declare.

## Authors’ contributions

CLY and LYH conceived the study and analyzed the data. CLY, JEW, GWC and DSC acquired the data. CLY drafted the manuscript, while critical revision was provided by JEW and DF. All authors read and approved the final version of the manuscript.

## References

[B1] Society for Healthcare Epidemiology of America; Infectious Diseases Society of America; Pediatric Infectious Diseases Society Policy statement on antimicrobial stewardship by the Society for Healthcare Epidemiology of America (SHEA), the Infectious Diseases Society of America (IDSA), and the Pediatric Infectious Diseases Society (PIDS) Infect Control Hosp Epidemiol 2012 33 322 327 22418625 10.1086/665010

[B2] Chung GW Wu JE Yeo CL Chan DS Hsu LY Antimicrobial stewardship: a review of prospective audit and feedback systems and an objective evaluation of outcomes Virulence In press 10.4161/viru.21626 PMC3654615 23302793

[B3] Yeo CL Chan DSG Earnest A Wu TS Yeoh SF Lim R Jureen R Fisher D Hsu LY Prospective audit and feedback on antibiotic prescription in an adult hematology-oncology unit in Singapore Eur J Clin Microbiol Infect Dis 2012 31 583 90 10.1007/s10096-011-1351-6 21845470

[B4] Poon LM Jin J Chee YL Ding Y Lee YM Chng WJ Chai LY Tan LK Hsu LY Risk factors for adverse outcomes and multidrug-resistant Gram-negative bacteremia in hematology patients with febrile neutropenia in a Singaporean university hospital Singapore Med J 2012 In press 23192498

[B5] Hersh AL Shapiro DJ Newland JG Polgreen PM Beekmann SE Shah SS Variability in pediatric infectious disease consultants’ recommendations for management of community-acquired pneumonia PLoS One 2011 6 e20325 10.1371/journal.pone.0020325 21655259 PMC3105054

